# Soft normed rings

**DOI:** 10.1186/s40064-016-3636-9

**Published:** 2016-11-10

**Authors:** Vakkas Uluçay, Mehmet Şahin, Necati Olgun

**Affiliations:** Department of Mathematics, Gaziantep University, 27310 Gaziantep, Turkey

**Keywords:** Soft sets, Normed rings, Soft normed rings, Soft normed ideal

## Abstract

Molodtsov introduced the concept of soft sets, which can be seen as a new mathematical tool for dealing with uncertainty. In this paper, we initiate the study of soft normed rings by soft set theory. The notions of soft normed rings, soft normed ideals, soft complete normed rings are introduced and also several related properties and examples are given.

## Background

Dealing with uncertainties is a major problem in many areas such as engineering, medical science, environmental science, social science et al. These kinds of problems can not be dealt with by classical methods, because classical methods have inherent difficulties. To overcome these kinds of difficulties, Molodtsov (1999) proposed a completely new approach, which is called soft set theory, for modeling uncertainty. Then Maji et al. (2003) introduced several operations on soft sets. Aktas and Cagman (2007) defined soft groups and obtained the main properties of these groups. Moreover, they compared soft sets with fuzzy sets and rough sets. Besides, Jun and Park (2008) defined soft ideals on BCK/ BCI-algebras. Feng et al. (2008) defined soft semiring, soft ideals on soft semiring and idealistic soft semiring. Sun et al. (2008) defined the concept of soft modules and studied their basic properties. Acar et al. (2010) defined soft rings and have introduced their initial basic properties such as soft ideals, soft homomorphisms etc. by using soft set theory. Shilov (1953) defined on decomposition of a commutative normed ring in direct sums of ideals. Gelfand et al. (1957) defined commutative normed rings. Freundlich (1949) introduced completely continuous elements of a normed ring. Raikov (1946) defined to the theory of normed rings with involution. Naĭmark (1964) defined normed rings. Shilov (1960) defined analytic functions in a normed ring. Jarden (2011) defined normed rings in 2011 and studied norms ‖.‖ of associative rings are generalizations of absolute values |.| of integral domains.

In this study, we define soft normed ring using soft sets. In “Preliminaries” section, well-known results of some preliminaries are given. In “Soft normed rings” section, a the notion of soft normed ring is given and various properties are studied. Finally “Conclusion” section presents the conclusion of our work.

## Preliminaries

In this section we recall some basic notations in soft set theory. Let *U* be an initial universe set and *E* be a set of all possible parameters under consideration with respect to *U*. The power set of *U* is denoted by *P(U)*. Molodstov defined the notation of a soft set in the following way:

### **Definition 1**

(Molodtsov 1999) A pair (*F, A*) is called a soft set over *U*, where *F* is a mapping given by $F:A\rightarrow P(U)$.

### **Definition 2**

(Maji et al. 2003) For two soft sets (*F*, *A*) and (*G*, *B*) over a common universe *U*, we say that (*F*, *A*) is a soft subset of (*G*, *B*) if

1. $$A\subseteq B$$ and
2. $$G(\varepsilon )\subseteq F(\varepsilon ),\forall \varepsilon \in B$$.

We write $(F,A)\subseteq (G,B)$. In this case (*G*, *B*) is called a soft superset of (*F*, *A*).

### **Definition 3**

(Maji et al. 2003) Let (*F*, *A*) and (*G*, *B*) be two soft sets over a common universe *U*. The union of (*F*, *A*) and (*G*, *B*) is defined as the soft set (*H*, *C*) satisfying the following conditions:

1. $$C=A\cup B$$,
2. $$\forall e\in C$$,
  $$H(e)= \left\{ \begin{array}{ll} F(e),&{\quad} if\,e\in A-B \\ G(e), &{\quad} if\,e\in B-A \\ F(e)\cup G(e), &{\quad} if\,e\in A\cap B \end{array} \right.$$

This is denoted by $(F,A)\,\tilde{\cup }\,(G,B)=(H,C)$.

### **Definition 4**

(Maji et al. 2003) Let (*F*, *A*) and (*G*, *B*) be two soft sets over a common universe *U*. The intersection of (*F*, *A*) and (*G*, *B*) is defined as the soft set (*H*, *C*) satisfying the following conditions:

1. $$C=A\cap B$$,
2. $$\forall e\in C$$,
  $$H(e)= \left\{ \begin{array}{ll} F(e),& \quad if\,e\in A-B \\ G(e), & \quad if\,e\in B-A \\ F(e)\cap G(e), & \quad if\,e\in A\cap B \end{array} \right.$$

This is denoted by $(F,A)\,\tilde{\cap }\,(G,B)=(H,C)$.

### **Definition 5**

(Das and Samanta 2012) Let *R* be the set of real numbers and *B*(*R*) be the collection of all non-empty bounded subsets of *R* and let *A* be taken as a set of parameters. Then a mapping $F:A\rightarrow B(R)$ is called a soft real set. If a soft real set is a singleton soft set, it will be called a soft real number and denoted by $\tilde{r}, \tilde{s},\tilde{t}$ etc.

$\tilde{0},\tilde{1}$ are the soft real numbers where $\tilde{0}(e)=0,\tilde{1}(e)=1 \ \ \forall e\in A$, respectively.

### **Definition 6**

(Das and Samanta 2012) Let $\tilde{r},\tilde{s}$ be two soft real numbers. Then the following statements hold:

1. $$\tilde{r}\le \tilde{s} \ \ if \ \ \tilde{r}(e)\le \tilde{s}(e) \ \ \forall e\in A$$,
2. $$\tilde{r}\ge \tilde{s} \ \ if \ \ \tilde{r}(e)\ge \tilde{s}(e) \ \ \forall e\in A$$,
3. $$\tilde{r}< \tilde{s} \ \ if \ \ \tilde{r}(e)< \tilde{s}(e) \ \ \forall e\in A$$,
4. $$\tilde{r}> \tilde{s} \ \ if \ \ \tilde{r}(e)> \tilde{s}(e) \ \ \forall e\in A$$.

### **Definition 7**

Jarden (2011) Let *A* be commutative ring with 1. An ultrametric absolute value of *A* is a function $||:A\rightarrow R$ satisfying the following conditions:

1. $$|a|\ge 0 \ \ and\ \ |a|=0 \Leftrightarrow a=0$$
2. There exists $a\in A$ such that $0<|a|<1$,
3. $$|a.b|=|a|.|b|$$,
4. $$|a+b|\le \max (|a|,|b|)$$.

## Soft normed rings

### **Definition 8**

Let *H* be an associative soft ring with $\tilde{1}$. A soft norm *H* is a function $\Vert .\Vert : H \rightarrow R(A)$ that satisfies the following conditions for all $\tilde{x}, \tilde{y} \in H$.

1. $$\Vert \tilde{x}\Vert \ge 0 \Leftrightarrow \tilde{x}=0$$; further $$\Vert \tilde{1}\Vert =\Vert -\tilde{1}\Vert =1$$.
2. There is an $\tilde{x}\in H$ with $0\,\tilde{<}\,\Vert \tilde{x}\Vert \,\tilde{<}\,1$,
3. $$\Vert \tilde{x}.\tilde{y}\Vert =\Vert \tilde{x}\Vert .\Vert \tilde{y}\Vert$$,
4. $$\Vert \tilde{x}+\tilde{y}\Vert \le \max (\Vert \tilde{x}\Vert ,\Vert \tilde{y}\Vert )$$.

The soft ring *H* with a soft norm ‖.‖ on *H* is said to be a soft normed ring and is denoted by $( H,\Vert .\Vert ,A)$ or $(H,\Vert .\Vert )$.

### *Example 1*

Let *R*(*E*) be the set of all soft real numbers. We define $\Vert .\Vert : R(E)\rightarrow R(A)$ by $\Vert \tilde{e} \Vert =|\tilde{e}| \ \ \forall e\in R(E)$, where $|\tilde{e}|$ denotes the module of the soft real number $\tilde{e}$. Then ‖.‖ satisfies all the soft norm axioms and so, ‖.‖ is a soft norm on *R*(*E*), and $(R(E),\Vert .\Vert ,E)$ is a soft normed ring.

### **Definition 9**

Let *H* be a soft normed ring. A soft sequence $\tilde{x}_{1}, \tilde{x}_{2},\ldots$ of soft elements of *H* is called soft Cauchy if for each $\varepsilon \tilde{>}\tilde{0}$ there exists $\tilde{m}_{0}$ such that $\Vert \tilde{x}_{n}-\tilde{x}_{m}\Vert \tilde{<}\, \varepsilon$ $\forall \tilde{m},\tilde{n}\,\tilde{\ge }\,\tilde{m}_{0}$. We say that *H* is soft complete if every Cauchy soft sequence converges.

### **Lemma 1**

*Let* *H* *be a soft normed ring and* $\tilde{x}, \tilde{y} \in H$. *Then*

1. $$\Vert-\tilde{x}\Vert =\Vert \tilde{x}\Vert$$
2. *If* $$\Vert \tilde{x}\Vert\,\tilde{<}\,\Vert \tilde{y}\Vert$$, *then* $$\Vert \tilde{x}+\tilde{y}\Vert =\Vert \tilde{y}\Vert$$.
3. *A soft sequence* $\tilde{x}_{1}, \tilde{x}_{2},\ldots$ *of soft elements of* *H* *is soft Cauchy if for each* $\varepsilon\,\tilde{>}\,\tilde{0}$ *there exists* $\tilde{m}_{0}$ *such that* $\Vert \tilde{x}_{m+1}-\tilde{x}_{m}\Vert\,\tilde{<}\,\varepsilon \ \ \forall \tilde{m}\,\tilde{\ge }\,\tilde{m_{0}}$.
4. *The soft map* $\tilde{x}\rightarrow \Vert \tilde{x}\Vert$ *from* *H* *to* *R*(*E*) *is soft continuous.*
5. *If* *H* *is soft complete then,* $\tilde{x}$ *soft series* $\sum _{n=0}^{\infty }\tilde{x}_{n}$ *of soft elements of* *H* *converges if and only if* $\tilde{x}_{n}\rightarrow \tilde{0}$.
6. *If* *H* *is soft complete and* $\Vert \tilde{x}\Vert \tilde{<}1$, *then* $1-\tilde{x}\in H^{*}$. *Moreover,* $(1-\tilde{x})^{-1} =1+\tilde{y}\ when\ \Vert \tilde{y}\Vert \tilde{<}1$.

### *Proof*

1. It is seen that $\Vert -\tilde{x}\Vert =\Vert -1\Vert .\Vert \tilde{x}\Vert =\Vert\,\tilde{x}\,\Vert$. Replacing $\tilde{x}\ by -\tilde{x}$ we get $\Vert \tilde{x}\Vert\,\tilde{\le }\,\Vert -\tilde{x}\Vert$ hence the claimed equality.
2. Suppose that $\Vert \tilde{x}+\tilde{y}\Vert =\Vert \tilde{y}\Vert$. Then by (i), $\Vert \tilde{y}\Vert =\Vert -\tilde{x}+(\tilde{x}+\tilde{y})\Vert\,\tilde{\le }\,max(\Vert -\tilde{x}\Vert , \Vert \tilde{x}+\tilde{y}\Vert )\,\tilde{\le }\,\Vert \tilde{y}\Vert$ which is a contradiction.
3. With $\tilde{m}_{0}$ in Lemma 1 let $\tilde{n}>\tilde{m}>\tilde{m_{0}}$. Then $\Vert \tilde{x}_{n} -\tilde{x}_{m}\Vert \tilde{<}max(\Vert \tilde{x}_{n} -\tilde{x}_{n-1}\Vert , \Vert \tilde{x}_{n-1}-\tilde{x}_{n-2}\Vert , \ldots ,\Vert \tilde{x}_{m+1}-\tilde{x}_{m}\Vert )\tilde{<} \varepsilon$
4. $\Vert \tilde{x}\Vert =\Vert (\tilde{x}-\tilde{y})+ (\tilde{y})\Vert\,\tilde{\le }\,max(\Vert \tilde{y}\Vert ,\Vert \tilde{x} -\tilde{y}\Vert )\,\tilde{\le }\,\Vert \tilde{x}-\tilde{y}\Vert +\Vert \tilde{y}\Vert$. Hence $\Vert \tilde{x}\Vert -\Vert \tilde{y}\Vert\,\tilde{\le }\,\Vert \tilde{x}-\tilde{y}\Vert$ and symmetrically $\Vert \tilde{y}\Vert -\Vert \tilde{x}\Vert\,\tilde{\le }\,\Vert \tilde{y}-\tilde{x}\Vert =\Vert \tilde{x}-\tilde{y}\Vert$. Therefore $|\Vert \tilde{x}\Vert -\Vert \tilde{y}\Vert\,|\tilde{\le }\,\Vert \tilde{x}-\tilde{y}\Vert$. Consequently the soft map $\tilde{x}\rightarrow \Vert \tilde{x}\Vert$ is a soft continuous.
5. Let $\tilde{s}_{n}= \sum _{i=0}^{\infty }\tilde{x}_{i}$. Then $\tilde{s}_{n+1}-\tilde{s}_{n}=\tilde{x}_{n+1}$. Thus by (iii) $\tilde{s}_{1}, \tilde{s}_{2},\ldots$ is a soft Cauchy sequence if and only if $\tilde{x}_{n}\rightarrow \tilde{0}$. Hence the soft series $\sum _{i=0}^{\infty }\tilde{x}_{n}$ converges if and only if $\tilde{x}_{n}\rightarrow \tilde{0}$.
6. It can be shown similarly as above.

### *Example 2*

Let *R*(*E*) be the space of all soft real functions that are defined and soft continuous on the interval [0, 1] with the soft norm given by

$$\Vert \tilde{x}\Vert =max_{0\tilde{\le }t\tilde{\le }1}|\tilde{x}(t)|.$$

Then *R*(*E*) is a soft normed ring (with the soft unit element $\tilde{x}(t)\equiv 1$) under ordinary soft multiplication (it is obvious that it satisfies all the conditions of Lemma 1)

### **Theorem 1**

*For any soft normed ring* *H*, *we can find a soft normed ring* $H^{'}$ *which is soft topologically and soft algebraically, soft isomorphic to* *H* *and such that*

### *Proof*

Every soft element $\tilde{x}$ of *H* generates a corresponding soft operator $T_{\tilde{x}}$ in terms of multiplication by $\tilde{x}: T_{\tilde{x}}\tilde{y}=\tilde{x}\tilde{y}$. This soft operator is soft linear. In the soft normed ring *Q*(*E*) of all soft linear operators soft mapping the soft Banach spaces H into itself, the soft operators $T_{\tilde{x}}$ form a soft subring $H^{'}$ with soft unit element (the soft unit element being the soft unit operator *E* generated by the soft unit element *e* of the soft ring *H*.)

Let us show that $H^{'}$ is a soft normed ring under the soft norm

$$\Vert T_{\tilde{x}}\Vert =sup_{\Vert \tilde{y}\Vert \le 1}\Vert \tilde{x}\tilde{y}\Vert.$$

In the proof we only require that $H^{'}$ is soft complete, i.e., $H^{'}$ is soft closed in *Q*(*E*). By the soft associativity of multiplication we have

$$T_{\tilde{x}}(\tilde{y}\tilde{z}) =\tilde{x}(\tilde{y}\tilde{z}) =(\tilde{x}\tilde{y})\tilde{z}=T_{\tilde{x}}\tilde{y}.\tilde{z}$$

It is not difficult to see that this property is characteristic for the soft operators of the soft ring $H^{'}$. If for a soft operator *T* and for arbitrary $\tilde{y}$ and $\tilde{z}$ the equation $T_{\tilde{y}\tilde{z}}=T\tilde{y}\tilde{z}$ holds, then, putting $T_{e}=\tilde{x}$, we have

$$T_{\tilde{y}}=T(e\tilde{y})=T_{e}.\tilde{y}=\tilde{x}\tilde{y}$$

i.e., *T* is the soft operator of multiplication by $\tilde{x}$.

Assume now that the soft operators $T_{n}\tilde{x}$ converge to $T\tilde{x}$ in the soft norm of the space *H* for every $x\in H$. By the soft continuity of multiplication with respect to the first factor, we then have:

$$T(\tilde{x}\tilde{y})=lim T_{n}(\tilde{x}\tilde{y})= lim T_{n}\tilde{x}.\tilde{y}=T \tilde{x}.\tilde{y}$$

And hence, by what we have just proved, *T* is also in $H^{'}$. Thus $H^{'}$ is soft closed in *Q*(*E*) not only in the sense of soft uniform convergence of operators, but also in that of soft strong convergence.

Obviously, the soft rings *H* and $H^{'}$ are soft algebraically soft isomorphic. Let us show that they are also soft topologically soft isomorphic. We have ($e\ne \tilde{0}$, and therefore $\Vert e\Vert\,\tilde{\ge }\,\tilde{0}$):

$$\Vert T_{\tilde{x}}\Vert =sup_{\Vert \tilde{y}\Vert \le 1}\Vert \tilde{x}. \tilde{y}\Vert\,\tilde{\ge }\,\Vert \tilde{x}.\frac{e}{\Vert e\Vert }\Vert = \frac{\Vert \tilde{x}\Vert }{\Vert e\Vert }$$

or

$$\Vert \tilde{x}\Vert\,\tilde{\le }\,\Vert e\Vert .\Vert T_{\tilde{x}}\Vert.$$

Thus the soft mapping $T_{\tilde{x}}\rightarrow \tilde{x}$ of the space $H^{'}$ onto the space *H* is soft continuous; but since both these spaces are soft complete, we have by the well-known soft Banach theorem, that the inverse soft mapping $\tilde{x}\rightarrow T_{\tilde{x}}$ is also soft continuous. We have thus proved that the soft rings *H* and $H^{'}$ are soft topologically isomorphic and, so, we have also proved the theorem since the soft norm in $H^{'}$ has the property $\Vert \tilde{x}.\tilde{y} \Vert\,\tilde{\le }\,\Vert \tilde{x}\Vert .\Vert \tilde{y}\Vert$ and $\Vert e\Vert =1$. Moreover we showed that every soft normed ring is soft topologically and soft algebraically soft isomorphic to a soft normed operator ring in a soft Banach space. $\square$

### *Remark 1*

If the condition $\Vert \tilde{x}.\tilde{y} \Vert\,\tilde{\le }\,\Vert \tilde{x}\Vert .\Vert \tilde{y}\Vert$ and $\Vert e\Vert =1$ is satisfied in the soft ring *H*, then *H* and $H^{'}$ are soft isometric. In this case, $\Vert \tilde{x}\Vert\,\tilde{\le }\,\Vert e\Vert .\Vert T_{\tilde{x}}\Vert$ gives $\Vert \tilde{x}\Vert\,\tilde{\le }\,\Vert T_{\tilde{x}}\Vert$. On the other hand, by $\Vert \tilde{x}.\tilde{y} \Vert\,\tilde{\le }\,\Vert \tilde{x}\Vert .\Vert \tilde{y}\Vert$ we have

$$\Vert T_{\tilde{x}}\Vert =sup_{\Vert \tilde{y}\Vert \le 1}\Vert \tilde{x}. \tilde{y}\Vert\,\tilde{\le }\,\Vert \tilde{x}\Vert sup_{\Vert \tilde{y}\Vert \le 1} \Vert \tilde{y}\Vert =\Vert \tilde{x}\Vert .$$

Combining these two inequalities, we obtain:

$$\Vert T_{\tilde{x}}\Vert =\Vert \tilde{x}\Vert.$$

### **Definition 10**

A soft set *F*(*I*, *A*) (or *I*(*A*)) of soft elements of a soft normed ring is called a soft normed ideal if it has the following properties:

1. If $\tilde{x} \in F(I,A)$ and $\tilde{y} \in F(I,A)$, then $\tilde{x}+\tilde{y}\in F(I,A)$;
2. If $\tilde{x} \in F(I,A)$, then $\tilde{z}.\tilde{x}\in F(I,A)\ \ \forall \tilde{z}\in R(A)^{*}$ A soft ideal *F*(*I*, *A*) of a soft normed ring *F*(*R*,*A*) is called a soft ideal if in addition;
3. $$F(I,A)\ne F(R,A)$$.

### *Example 3*

Let *H* be a soft normed ring. Then each of the following families is a soft normed ideal over H with the same set of parameters *A*,

1. $$F(I,A)=\{\emptyset \}$$
2. $F(I,A)=\{F(I,A):F(I,A)$ *is a soft set over H with the fixed set of parameters A*}.

## Conclusion

Normed rings have previously been described in the classical sense. In this study, normed rings are defined on soft sets for the first time. This may lead to an ample scope on soft normed rings in the soft set setting. In this paper, we defined a soft normed ring. We then investigated some related properties and some theorems. To extend this work, one can study the properties of soft normed rings in other algebraic structures and fields.
